# Phages versus Antibiotics To Treat Infected Diabetic Wounds in a Mouse Model: a Microbiological and Microbiotic Evaluation

**DOI:** 10.1128/mSystems.00542-20

**Published:** 2020-11-10

**Authors:** Jean-François Huon, Emmanuel Montassier, Anne-Gaëlle Leroy, Matthieu Grégoire, Marie-Anne Vibet, Jocelyne Caillon, David Boutoille, Dominique Navas

**Affiliations:** a CHU Nantes, Clinical Pharmacy Unit, Nantes, France; b EA3826, Laboratory of Clinical and Experimental Therapeutics of Infections, Université de Nantes, Nantes, France; c Microbiotas Hosts Antibiotics Bacterial Resistances (MiHAR), Université de Nantes, Nantes, France; d CHU Nantes, Department of Emergency Medicine, Nantes, France; e CHU Nantes, Department of Bacteriology, Nantes, France; f Clinical Pharmacology Department, Nantes University Hospital, Nantes, France; g UMR INSERM 1235, The Enteric Nervous System in Gut and Brain Disorders, University of Nantes, Nantes, France; h DRCI, Plateforme de Méthodologie et de Biostatistique, CHU Nantes, Nantes, France; i CHU Nantes, Department of Infectious Diseases, CIC UIC 1413 INSERM, University Hospital, Nantes, France; Teagasc Food Research Centre

**Keywords:** bacteriophages, microbiota, wound

## Abstract

Diabetes is marked by a range of complications, including chronic infections that can lead to limb amputation. The treatment of infected wounds is disrupted by arteriopathies that reduce tissue perfusion as well as by the critical development of bacterial resistance. We evaluated the impact of a local application of bacteriophages compared to that of a *per os* administration of amoxicillin-clavulanic acid in a mouse model of Staphylococcus aureus wound infection. We found that phage treatment resulted in improved clinical healing and a reduction in local bacterial load at 7 and 14 days postinfection. Unlike antibiotics, phage therapy did not deplete the intestinal microbiota of treated animals. Amoxicillin resulted in a reduction of alpha and beta diversities of the murine microbiota and disturbed architecture even 7 days after the end of treatment, whereas phage treatment did not impinge on the microbiota.

**IMPORTANCE** The management of diabetic foot infections is frequently a dead end for surgeons and infectious disease specialists. When the pathogen to be treated is not resistant to conventional antibiotics, the latter tend to unbalance the intestinal microbiota, which is linked to multiple pathologies. A local treatment with bacteriophages, in addition to being as much or even more effective than antibiotics from a clinical and microbiological point of view, makes it possible to respect the patient’s microbiota. These results suggest that the use of this therapeutic alternative is a major avenue and that the introduction of recommendations for their use is now necessary.

## Abstract (precis)

The management of diabetic foot infections is frequently a dead end for surgeons and infectious disease specialists. When the pathogen to be treated is not resistant to conventional antibiotics, the latter tend to unbalance the intestinal microbiota, which is linked to multiple pathologies. A local treatment with bacteriophages, in addition to being as much or even more effective than antibiotics from a clinical and microbiological point of view, makes it possible to respect the patient’s microbiota. These results suggest that the use of this therapeutic alternative is a major avenue and that the introduction of recommendations for their use is now necessary.

## INTRODUCTION

One of the most dreaded complications of diabetes, deriving from the intricated consequences of neuropathy and angiopathy, is diabetic wound, with an estimated lifetime risk of 15% (1). As the healing capacities of these patients are diminished or even abolished, chronic infections may establish, frequently leading to lower-limb amputation and increased morbidity, with a very significant societal cost (2). Many bacterial species can be found in these ulcers, but the most common by far is Staphylococcus aureus, with a prevalence of up to 42% in diabetic foot ulcers (3).

The lack of tissue perfusion in diabetic patients due to macro- and microangiopathy, while leading to limb necrosis, hinders the action of active antibiotics against the bacteria responsible for chronic wound infections. In addition, antibiotics can have potentially harmful side effects, as their overuse has been linked to microbiota impairment and related disorders (4). Lastly, while the pharmaceutical industry’s interest in the search for new antibiotics has diminished, innovative solutions are urgently required.

Bacteriophages are part of the arsenal of therapies developed against refractory infections. These bacterial viruses that kill their host have long been used for treating all types of infections, including serious and life-threatening ones (5). Their ability to target specifically one type of pathogenic bacteria and their proven safety (6) support their use in multiple indications, especially in wound infection. The external use of phages to treat localized infections was showed to be effective in different animal models (7–13). However, it remains important to evaluate all the possibilities in terms of dosage, rhythm of administration, and association with antibiotics and to assess the impact of topically administered phages on the intestinal microbiota, given that no studies have been found in the literature on this subject.

Hence, our work was developed to assess the impact of a topical application of phages, administered alone or in combination with oral amoxicillin-clavulanic acid, in a mouse model of chronic S. aureus-infected diabetic wound.

## RESULTS

### Plasma concentrations of amoxicillin in treated mice.

**FIG 1:**
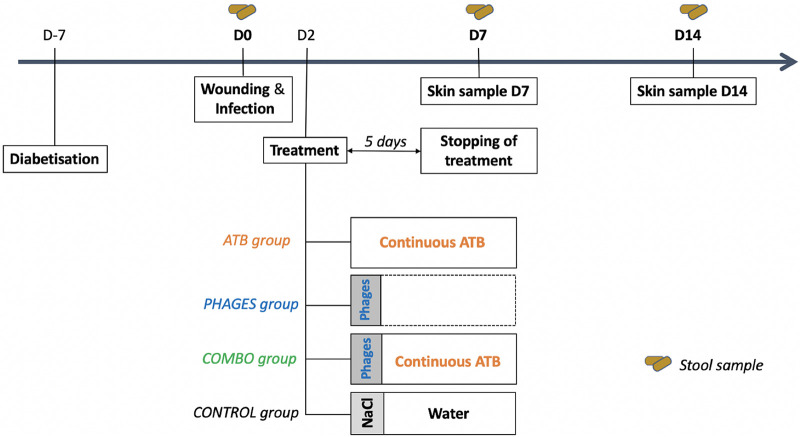
Diagram of the therapeutic strategy. Amoxicillin-clavulanic was administered at a dosage of 60 mg/day *per os* for 5 days, and phages were administered through a unique local administration. ATB, amoxicillin plus clavulanic acid; combo, combination of antibiotics and bacteriophages.

Samples collected from several mice treated with antibiotic (ATB) (Fig. 1) after 5 days yielded a mean of 14.59 (interquartile range[IQR], 6.56) mg/liter plasma amoxicillin in treated mice (*n* = 18), which corresponds to an equilibrium plasma concentration of approximately 30 times the MIC, with 100% of the time greater than 6× MIC.

### Overall survival.

**FIG 2:**
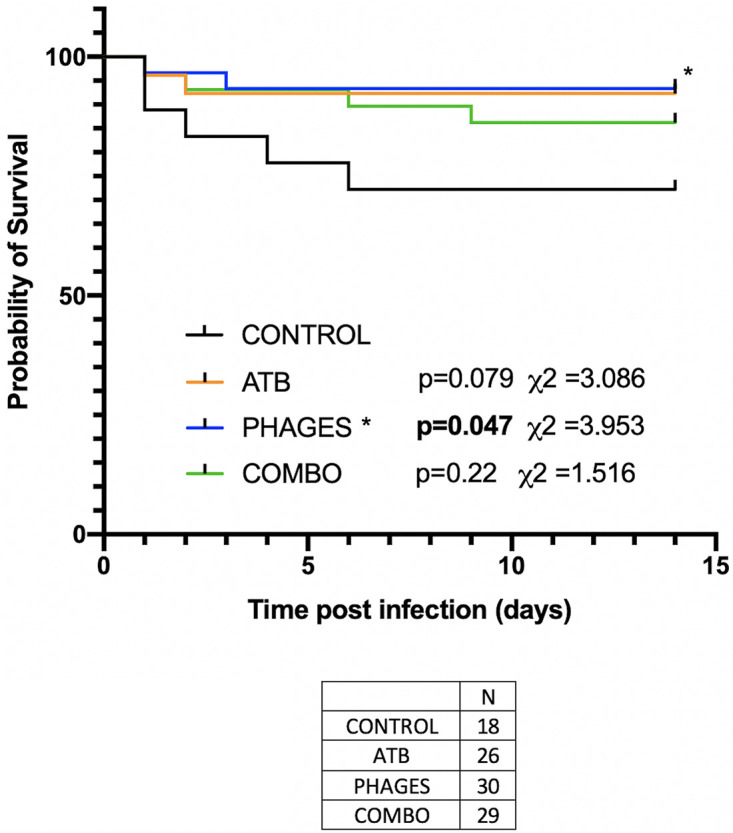
Overall survival. Kaplan-Meier survival curve for overall survival. Black line represents control mice. Blue line represents survival of phage-treated mice, orange line represents survival of ATB-treated mice, and green line represents combo-treated mice. Number of mice: ATB, 26; phages, 30; combo, 29; control, 18.

The estimated 14-day overall survival of control mice was 73% (Fig. 2). Comparatively, the overall survival of ATB-, phage-, and combo (phage and antibiotic)-treated mice appeared to be improved to 92%, 93%, and 86%, respectively, with a significant difference for the phage-treated group (*P* = 0.047).

### Clinical impact.

**FIG 3:**
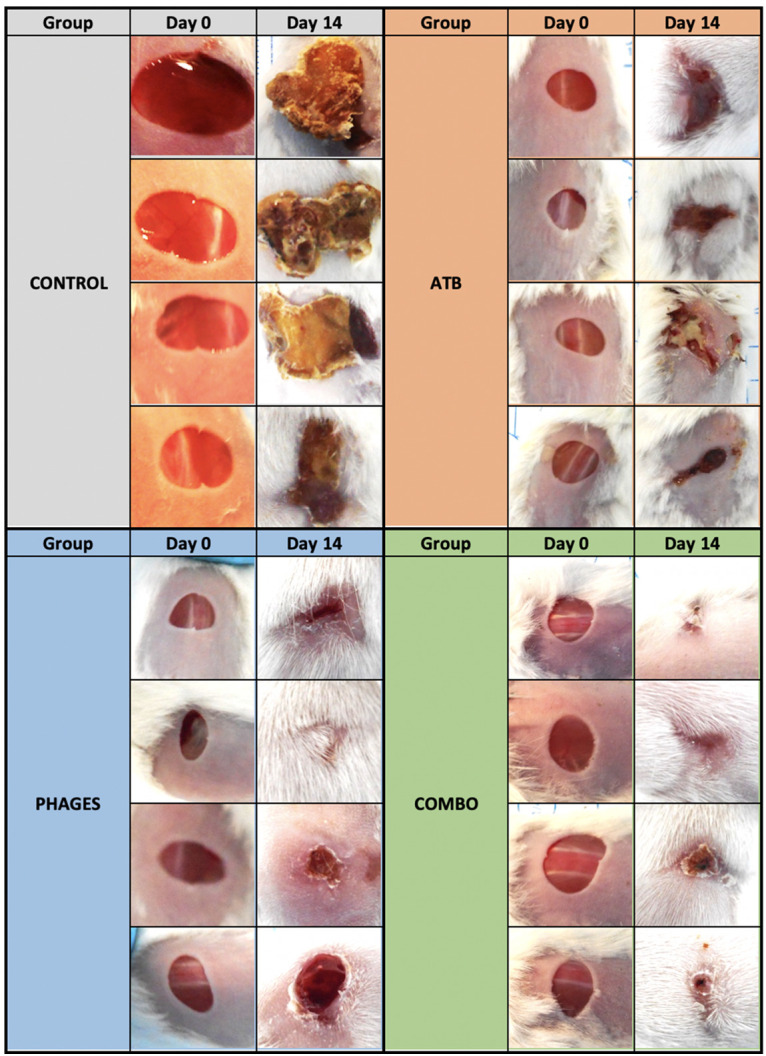
Evolution of wounds in each group (control or treated with ATB, phages, or combo) at days 0 and 14. In each group, each line of photos illustrates the evolution on the same animal.

By day 14, the evolution of the wound was unfavorable in the control group, which showed wound enlargement, nonhealing, and purulence (Fig. 3). The wounds in mice treated with bacteriophages (alone or in combination with ATB) had progressed favorably compared to the wound evolution in the control group. Wounds in mice treated only with ATB improved more slowly (delayed healing) and sometimes appeared purulent.

### Microbiological impact.

Bacterial loads in the wounds of phage-treated mice, in both the “phages” and “combo” groups, were significantly lower than in the control group and in the ATB group at days 7 and 14 (Table 1).

**TABLE 1:** Bacterial loads at day 7 and day 14 of infection in skin samples

Group^*a*^	CFU/g tissue		*P* value	CFU/g tissue		*P* value
	Day 7	Difference from control		Day 14	Difference from control	
Control (*n* = 33)	9.40 ± 0.69			8.20 ± 1.50		
ATB (*n* = 24)	8.54 ± 0.88	−0.86	0.002	7.70 ± 1.19	−0.5	0.2
Phages (*n* = 25)	6.92 ± 0.50	−2.48	3.85 × 10^−8^	5.42 ± 2.03	−2.28	7.8 × 10^−4^
Combo (*n* = 24)	7.34 ± 0.87	−2.06	1.26 × 10^−6^	6.42 ± 1.75	−1.28	0.02

a *n*, number of animals in each group.

Within the phages group samples, no bacterial development was observed in 3 of 13 samples at day 14. Finally, the differences in wound bacterial loads of the ATB, phages, and combo groups, compared to that in the control group, were all significant except for the ATB group at day 14. There was no difference in efficacy between the phages and combo groups at either day 7 (*P* = 0.31) or day 14 (*P* = 0.23). On the other hand, a significant difference was found between the samples from the ATB and phages groups at day 7 (*P* = 4.2 × 10^−4^) and day 14 (*P* = 0.07).

### Microbial community analysis.

**FIG 4:**
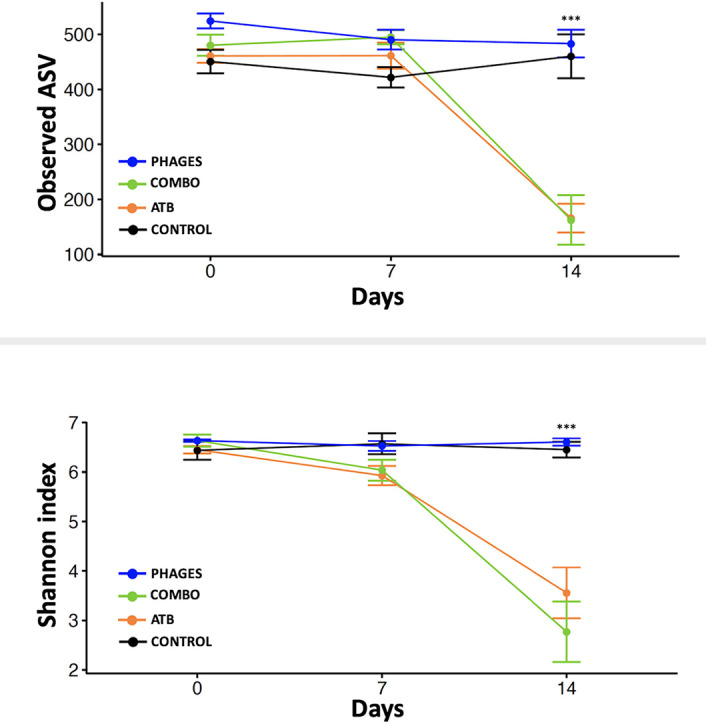
Alpha diversity indices in samples collected from ATB-, combo-, or phage-treated mice and control mice. Analyses were performed on 16S rRNA gene V4 region data.

**(i) Intestinal microbiota diversity is altered in mice receiving antibiotic alone or associated with phages but not in mice receiving phages alone.** Using non-phylogeny- and phylogeny-based alpha diversity metrics, we did not find a significant difference between the 4 groups of mice at day 0 (Kruskal-Wallis test, Shannon index, *P* = 0.24; observed amplicon sequence variants [ASV], *P* = 0.06) or at day 7 (Kruskal-Wallis test, Shannon index, *P* = 0.06; observed ASV, *P* = 0.12) (Fig. 4). We found a significant difference in alpha diversities between the 4 groups of mice at day 14 (analysis of variance [ANOVA], Shannon index, *P* < 0.001; observed ASV, *P* < 0.001), with decreased diversity in ATB and combo groups (Dunn test, observed ASV, *P* = 0.02 and 0.01, respectively; Shannon index, *P* = 0.003 and 0.02, respectively) (Fig. 4). Moreover, alpha diversity decreased significantly between day 7 (D7) and day 14 (D14) in both ATB and combo-treated mice (linear mixed-effects model, *P* < 0.001 and *P* < 0.001, respectively), but not in phage-treated mice (linear mixed-effects model, *P* = 0.95), showing that intestinal microbiota diversity did not return to pretreatment level 7 days after antibiotic discontinuation.

**FIG 5:**
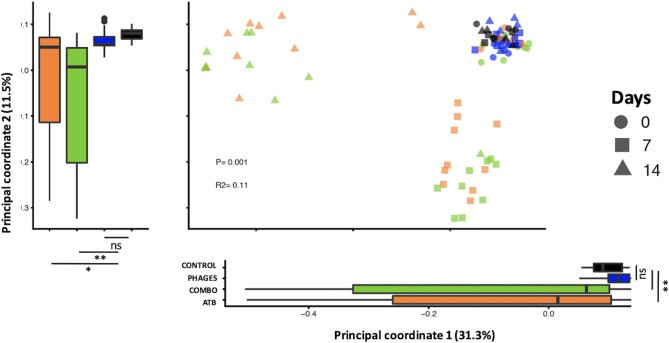
Beta diversity comparisons of the gut microbiomes of the fecal samples collected from ATB-, combo-, or phage-treated mice and control mice. Analyses were performed on 16S rRNA gene V4 region data. Principal-coordinate analysis of unweighted UniFrac distances. Proportions of variance explained by each principal-coordinate axis are denoted in the corresponding axis labels.

**FIG 6:**
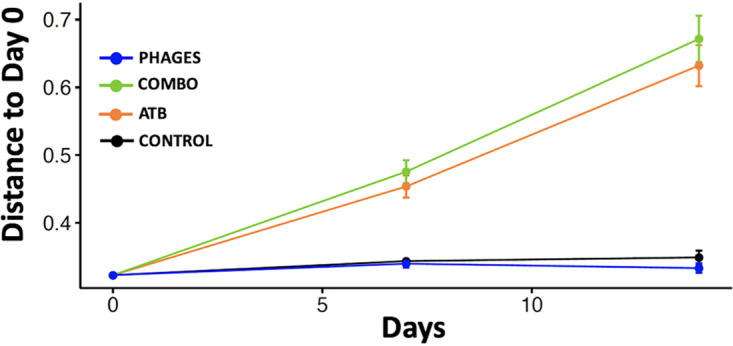
Beta diversity of unweighted UniFrac distance variations from pretreatment samples.

A principal-coordinate analysis of the unweighted UniFrac distances showed a significant change in the phylogenetic diversity in mice receiving ATB or combo treatment compared to that of control and mice that received phages (permutational multivariate analysis of variance [PERMANOVA], *r*^2^ = 0.11, *P* value = 0.01) (Fig. 5). Phylogenetic diversity in fecal samples collected at day 7 in mice that received ATB or combo treatment changed along principal coordinate 2 (Dunn test, *P* < 0.001 or *P* < 0.001, respectively, compared to that in control and mice that received phages), and phylogenetic diversity in fecal samples collected at day 14 in mice that received ATB or combo treatment changed along principal coordinate 1 (Dunn test, *P* < 0.003 or *P* < 0.004, respectively, compared to that in control and mice that received phages). When comparing unweighted UniFrac distances at day 7 and day 14 to pretreatment samples, we observed a significant difference in phylogenetic diversity in mice that received ATB or combo treatment at day 7 (linear mixed-effects model, *P* value = 0.007 or *P* value = 0.002, respectively) and at day 14 (linear mixed-effects model, *P* value = 0.003 or *P* value = 0.001, respectively), showing that intestinal microbiota architecture is still disrupted 1 week after antibiotic discontinuation (Fig. 6).

**FIG 7:**
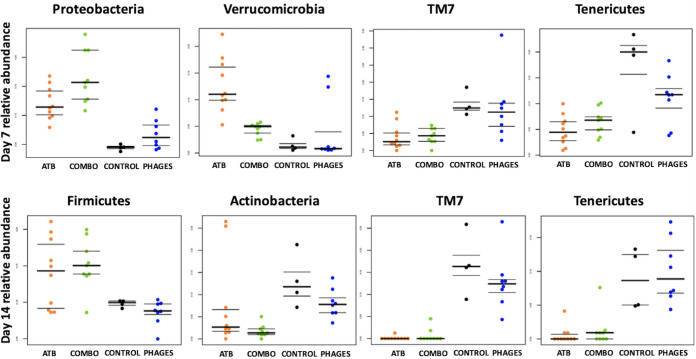
Relative abundances of the most significant phyla that were significantly different between the 4 groups of mice at day 7 and day 14 using ANOVA.

**FIG 8:**
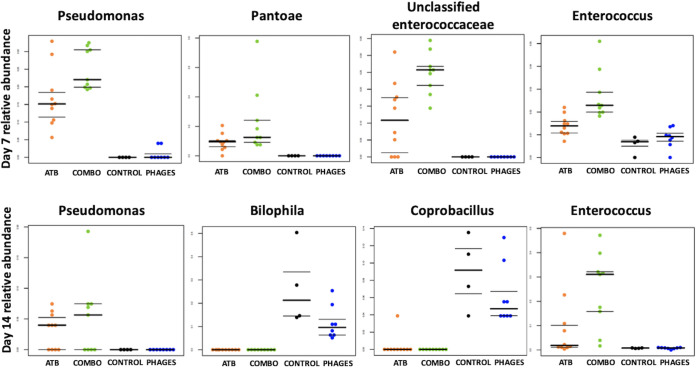
Relative abundances of the most significant genera that were significantly different between the 4 groups of mice at day 7 and day 14 using ANOVA.

**FIG 9:**
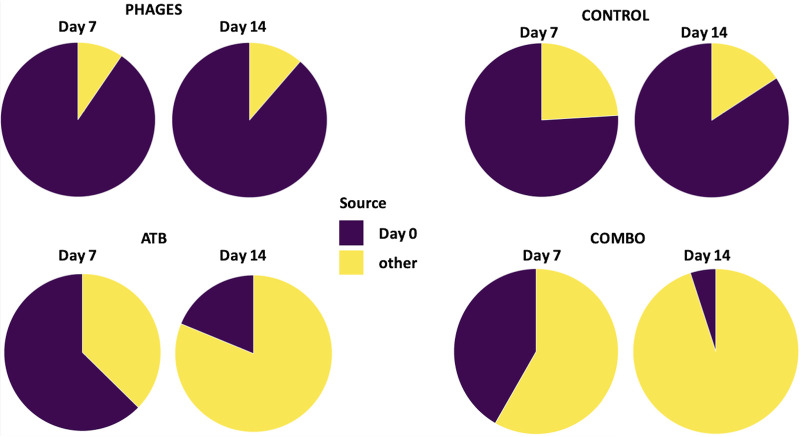
SourceTracker was used to estimate the proportions of pretreatment samples (source communities) that contribute to the constitution of samples collected on day 7 and day 14 (sink communities).

**(ii) The microbiota of mice receiving antibiotics alone or associated with phages were enriched in *Verrucomicrobia* and *Proteobacteria* and depleted in *Tenericutes* and TM7.** We then explored taxonomic changes between the 4 groups of mice. At the phylum level, we did not find any significant difference between the groups at day 0. At day 7, we found a significant enrichment in *Proteobacteria* and *Verrucomicrobia* and a significant depletion in TM7 and *Tenericutes* (ANOVA, false-discovery rate [FDR]-corrected *P* value < 0.05) in mice that received ATB or combo treatment compared to that in control and phage-treated mice (Fig. 7). At day 14, we found a significant enrichment in *Firmicutes* and a significant depletion in *Actinobacteria*, TM7, and *Tenericutes* (ANOVA, FDR-corrected *P* value < 0.05) in mice that received ATB or combo treatment compared to that in control and phage-treated mice (ANOVA, FDR-corrected *P* value < 0.05). At the genus level, the relative abundances of two genera were significantly different between the 4 groups at day 0; 59 genera among 229 (26%) were significantly different at day 7 (including *Pseudomonas*, *Pantoea*, and *Enterococcus*, which were increased in ATB or combo mice) (Fig. 8), and 36 (16%) were significantly different at day 14 (including *Pseudomonas* and *Enterococcus*, which were increased in mice treated with antibiotic or antibiotic plus phages) (Fig. 8). Moreover, SourceTracker was used to assess whether intestinal microbiota from samples collected on day 7 and day 14 in the 4 groups (sink communities) were mostly similar to intestinal microbiota from pretreatment samples (source communities) (Fig. 9). Based on this algorithm, we found that intestinal microbiota collected on day 7 and day 14 in mice treated with phages mainly originated from pretreatment samples, whereas the proportion of pretreatment intestinal microbiota was minor in samples collected on day 7 and day 14 in mice that received ATB or combo treatment.

## DISCUSSION

Our work demonstrated the potential effectiveness of an external single use of specific phages on survival, clinical outcome, and bacterial loads in a mouse model of S. aureus-infected diabetic wound compared or associated with an oral antibiotic treatment. Phage treatment also demonstrated a low impact on the intestinal microbiota in comparison to that of the antibiotic treatment.

We chose to use as a reference comparator the *per os* administration of the combination amoxicillin-clavulanic acid. According to the 2006 French-speaking Infectious Disease Society consensus, this antibiotic is recommended as first-line therapy in diabetic foot infections (excluding osteitis) due to methicillin-susceptible S. aureus (MSSA). In the case of superficial or recent infection, treatment can be initiated *per os* on an outpatient basis. Gariani et al. also demonstrated in 2019, in a retrospective study of 794 diabetic foot infections, that *per os* amoxicillin-clavulanic acid was a reasonable option for this indication (14). In our model, high drinking water consumption by diabetic mice with polydipsia allows us to mimic continuous administration of the antibiotic, as confirmed by plasma pharmacokinetic samples.

We chose to administer two volume-to-volume mixed bacteriophages with a narrow spectrum of activity, specific to our clinical strain. Indeed, due to the risk of emergence of mutants, broad-spectrum phages, therefore, less specific, are used in truly extreme cases (15).

Our study demonstrated that a single application of specific bacteriophages made it possible to reduce the bacterial load of the wound by more than 2 log CFU/g at days 7 and 14.

Administration of amoxicillin-clavulanic acid alone resulted in a decrease of 0.86 and 0.5 log CFU/g at days 7 and 14, respectively, compared to that in the control group. These data are consistent with various publications, as in the study by Shivaswamy et al. in which diabetic rats were infected at the wound site with a strain of A. baumannii and then treated at 48 h with either a multiplicity of infection (MOI) of 10 phages or intramuscular colistin injection (12). Six to 8 days later, the bacterial load decreased by 1 log CFU/g in the antibiotic-treated group compared with an average of 7 log CFU/g in the phage group. This result, which is higher than ours, is debatable, as the number of animals used is not specified, and null values that do not take into account the detection limit are found in this work (12).

It should be noted that on day 16, the bacteriological skin samples from the mice in the study by Shivaswamy et al. were all sterile, regardless of the treatment arm. This was not the case in our study at day 14, which could be explained by the fact that the animal model, the bacterial strain, and the antibiotics were different between the two studies but also by the fact that the MOI used in our study was 10,000 times lower than that used in the study by Shivaswamy et al. (12). Indeed, we used an MOI of 0.001 compared to an MOI of 10 in their work.

The same team also studied the impact of a local spray of bacteriophages on a wound infected with S. aureus in diabetic rats (12). The results of this study are in line with our results, showing a better efficacy of local phage treatment on an infected wound than oral clindamycin administration.

Very few studies have evaluated the efficacy of topical phages in a murine diabetic model of infected wounds. However, Mendes et al. showed that local administration of a cocktail of phages specific to three bacterial strains (S. aureus, P. aeruginosa, and A. baumannii) in diabetics rats wounds resulted in a decrease of approximately 3 log CFU/g within 1 day and that this efficacy persisted at day 4 (11). Improved wound healing was also demonstrated clinically and histologically. Their administration protocol was different from ours: a phage loading dose was applied for the first 24 h (100 μl of the cocktail every 4 h) and then continued for 4 days, twice daily. So, while we proceeded with a single administration, we think that multiplying the applications in our model could still improve the bacteriological efficiency of our treatment.

In our work, coadministration of phages and antibiotics was not shown to be more effective than the use of phages alone. Indeed, although we noticed the same clinical impact on wound healing, the bacterial load in the wound was lower after treatment when the wound was managed with phage therapy alone. These results are not consistent with some of the literature, in which the combination of antibiotics and phages increased the effectiveness of treatment. For example, Kirby's study showed that the combined use of gentamicin and phage SA5 was more effective than each alone on a continuous culture of S. aureus (16). Rahman et al. showed in 2011 that coadministration of the SAP-26 phage with rifampicin had a greater effect on the eradication of S. aureus biofilm *in vitro* than either the phage or the antibiotic alone (17). This is also true in the mouse model of diabetic foot by Chhibber et al. (18). Maximum reduction in bacterial titer was achieved when the MR-10 phage was combined with linezolid. Another work showed that combining phages with sublethal concentrations (below the MIC) of some antibiotics can increase phage production by the bacterial host, describing this phenomenon as “phage antibiotic synergy” (PAS) (19, 20). This synergistic mechanism is thought to be related to cell elongation of the bacteria by the antibiotics (by altering wall integrity), promoting phage replication, and possibly external attachment to the bacteria due to an increase in cell surface area (21).

We had expected such a synergy in our study, especially in view of the high doses of amoxicillin-clavulanic acid administered. Lopes et al. explain in their work that this could be due to the concentration of the antibiotic as well as the delay between the administration of the phage and the antibiotic (22). Lethal and non/sublethal concentrations would inhibit bacterial replication and thus simultaneously inhibit phage replication. Thus, Lopes et al. added that treating at an MOI of 100 rather than 1 at the initiation of treatment could circumvent this problem (22). Our work was conducted at an MOI of 0.001, which would explain why the synergy sought by the combination finally became not different.

In addition, several studies have shown that a reverse mechanism may exist. In specific cases that remain to be defined, some antibiotics could have an antagonistic effect on the action kinetics of phages, such as ciprofloxacin on Escherichia coli (23). It is in this sense that Tagliaferri et al. conclude that “negative interference [of antibiotics with phages] might be more common [than] as assumed, and it is possible that such experimental outcomes in the laboratory are less frequently reported than the positive ones” (24).

A limit of our work is not having measured the number of bacteriophages in tissues over time to understand the kinetics of the interactions.

The devastating effect of antibiotics on gut microbiota is a major concern (4). Indeed, antibiotic treatment has been shown to dramatically decrease microbiota taxonomic richness and diversity (25–27). Lower diversity significantly reduces ecological stability and resistance to pathogen, increasing the susceptibility to infection and diarrhea (28). Here, we confirmed previous findings showing enrichment in *Proteobacteria*, naturally or frequently resistant to amoxicillin, including unclassified *Enterobacteriaceae*, *Pantoea*, and *Pseudomonas* in the gut microbiome following antibiotic treatment (29). In our study, we confirm that the intestinal microbiota alteration persisted 1 week after antibiotic discontinuation (30, 31). Knecht et al. reported that *Enterococcus* indicated treatment with ampicillin/sulbactam (32), and Ferrer et al. also found that *Enterococcus* promptly expanded after the administration of the same treatment (33). Yin et al. reported an increase in *Pseudomonas* following antibiotic treatment (34), and Vrieze et al. also reported the same trend (35). Moreover, antibiotic treatment may increase the resistance gene repertoire in the gut, leading to multidrug-resistant infections (36, 37).

One of the fears of administering bacteriophages is that despite the natural presence of commensal phages within the human microbiota, therapeutic phages will always act as an external agent (38). Here, we showed that topical application of bacteriophages did not alter the intestinal microbial community. While some authors state that phages given orally induce minimal changes in the phylogenetic composition of the microbiota (39), others show that this therapy substantially alters the composition of the murine intestinal microbiota (40). A recent study showed that there were no differences in taxon abundance and phylogeny in the gut microbiome of phage-treated and control pigs (*n* = 17) after 14 days of oral treatment (41). For the latter, phage-induced bacterial modulation impacts the intestinal metabolome and results in effects on nontarget species. However, the phages used in these publications targeted bacteria naturally present in the intestinal microbiota (*Clostridium*, *Helicobacter*, *Prevotella*, *Escherichia*, *Salmonella*, etc.). As Staphylococcus aureus is not a common pathogen of the digestive tract, it is consistent that the application of our bacteriophage preparation had no impact on the intestinal microbiota of treated animals. Nevertheless, microbiota studies to date have been conducted on oral phage delivery models. Our results concerning the impact of topical administration of bacteriophages tend to demonstrate that this route of administration allows for a quantitative and qualitative respect of the intestinal microbiota. Indeed, phylum and genus analyses showed no impact compared to the control groups. This is in line with the results of Febvre et al. that showed there were no significant changes to alpha and beta diversity parameters, suggesting that consumed phages did not globally disrupt the microbiota (42).

### Conclusion.

The local use of bacteriophages to treat an infected chronic diabetic wound appears to be an effective alternative to conventional oral antibiotic treatment. Compared to treatment with systemic amoxicillin-clavulanic acid, bacteriophages appear to have a superior clinical and microbiological impact. In addition, and in contrast to antibiotics, the minor impact of phages on the intestinal microbiota is an advantage that needs to be taken into account in current anti-infective strategies. The integration of this new anti-infective class needs to be discussed and organized to facilitate its use in the future.

## MATERIALS AND METHODS

### Bacterial strain.

A clinical strain of S. aureus (NSA1385) isolated from a diabetic foot wound, susceptible to methicillin and amoxicillin-clavulanic acid (MIC of 0.5 mg/liter), was provided by Jean-Philippe Lavigne (University of Nîmes, France). The NSA1385 strain was grown overnight in brain heart infusion broth at 37°C (Becton, Dickinson, Franklin Lakes, NJ). Immediately before use, the bacterial pellet (centrifuged at 800 × *g* for 10 min) was washed twice using 0.9% NaCl. After the second wash, the pellet was resuspended in sterile saline, and the inoculum was calibrated by nephelometry.

### Antibiotic.

One gram of amoxicillin and clavulanic acid (amoxicillin/clavulanic acid injection, 1 g/200 mg; Sandoz Laboratories) was diluted in a 250-ml bottle of drinking water. The antibiotics were replaced every 12 h with a newly reconstituted solution.

### Bacteriophages.

From its biocollection, the Pherecydes Pharma laboratory selected two phages, named PN1815 and PN1957, as the most efficient on the clinical strain studied. These phages were isolated from raw sewage. Both were classified within the *Caudovirales* order. PN1815 is in the family *Myoviridae* and PN1957 us in the family *Podoviridae*. The two suspensions were mixed at equivalent volumes and extemporaneously at the time of administration.

### Ethics statement.

All experiments were approved by the French Ministry of Higher Education and Research (authorization numbers 6750 and 15029) and locally by the animal ethics committee of the Health Research Institute. Furthermore, all experiments were in accordance with the European Directive 86/609/EC (43) and the Guide for the care and use of laboratory animals (44). Due to the polydipsia, polyphagia, and polyuria of the diabetic mice, weights were monitored closely and bedding was changed more frequently.

### Animals.

Ten-week-old female Swiss mice (RjOrl/SWISS; Janvier Laboratory, Saint-Berthevin, France) weighing approximately 30 g were maintained on a 12-h light/dark cycle and had access to food and water *ad libitum*. Their well-being was checked daily.

### Infected diabetic wound model.

Infected diabetic wound was induced as previously described (45). The animal model of type 1 diabetes mellitus was established by injecting streptozotocin intraperitoneally at a dose of 170 mg/kg body weight (46). Diabetes mellitus (nonfasting plasma glucose concentrations > 300 mg/dl) was confirmed by regular tail vein blood glucose monitoring. Diabetic mice were shaved, and a 6-mm wound was made on the top of their back. Chronification of the wounds was obtained by the administration of mercaptosuccinic acid locally (150 mg/kg body weight) and 3-amino-1,2,4-triazole by the intraperitoneal route (1 g/kg body weight) (47). Each wound was inoculated with 1 × 10^8^ CFU/ of strain NSA1385 in 200 μl of phosphate-buffered saline (PBS). The animals were then placed in individual cages to avoid cross-contamination. The infection was allowed to establish for 48 h.

### Treatments.

The mice were randomly divided into four therapeutic groups of animals (Fig. 1) after wounding and infection at day 0. Each group was treated 48 h after infection. The antibiotic (ATB) group was challenged with *per os* amoxicillin-clavulanic acid solution administered via drinking water for 5 days at an amoxicillin dosage of 60 mg a day as determined by preliminary studies (data not shown). The phages group received a single local administration of 200 μl of a 10^5^-PFU/ml phage suspension over the wound and under the edges. This dosage allowed us to obtain the required multiplicity of infection (MOI) of 0.001. The combo group received both treatments simultaneously: local phage administration 48 h after infection and 5 days of *per os* antibiotic treatment. Lastly, the control group received a single administration of 200 μl of saline solution and standard drinking water.

The mice were killed either on day 7 (end of the treating period) or on day 14 (7 days after the end of the treating period) by carbon dioxide euthanasia.

### Plasmatic antibiotic concentration monitoring.

Plasmatic amoxicillin concentrations were monitored using high-performance liquid chromatography (HPLC) on day 5 of treatment when the animals were euthanized. Samples were stored at −80°C until measurement.

### Overall survival.

To determine overall survival, endpoints beyond which we terminated the pain of the animals via euthanasia were chosen. Euthanasia was decided when the animal reached a score based on behavioral (activity, posture, and aggression) and pathophysical (hair, orbital tightening, respiration rate, and weight loss) indicators. Overall survival after infection was estimated using the Kaplan-Meier method and compared to overall survival of control mice (infected but not treated). The numbers of mice per group were 18, 26, 30, and 29 for the control, ATB, phages, and combo groups, respectively.

### Clinical evolution.

Photographs of the wounds were taken on days 1 and 14 to assess wound healing. Changes in wound size, pus, and scabbing were noted. An increase in wound size and pus production was considered unfavorable.

### Bacterial counts.

Wound bed and edge tissues were excised using surgical scissors. Tissues were weighed and homogenized in 0.5 ml of saline buffer, and dilutions were prepared. Fifty microliters of these solutions was used for quantitative bacterial cultures by a spiral plating system on Chapman agar and chromogenic agar plates, incubated at 37°C. The bacterial count was performed after 48 h of incubation. The numbers of mice per group were 33, 24, 25, and 24 for the control, ATB, phages, and combo groups, respectively.

### Microbial community analysis.

**(i) Bacterial 16S rRNA gene amplification and sequencing.** On days 0, 7, and 14, stool samples were taken in order to measure the impact of the different therapeutic regimens on the intestinal microbiota of the model. The mice were placed for 30 min in individual empty cages, and two feces samples were collected, transferred to an Eppendorf tube, and frozen at −80°C. For each of the 3 days, 4 samples were taken for the control group, 10 for the ATB group, 8 for the phages group, and 9 for the combo group. Fecal pellets were collected from mice during week 16 of life and submitted to the University of Minnesota Genomics Center for DNA extraction, generation of 16S amplicons, and sequencing. Briefly, DNA was extracted using the PowerSoil DNA isolation kit, followed by amplification of the V4 region of the 16S rRNA gene using standard methods (48). DNA libraries were generated from the resulting amplicons using the Illumina TruSeq Nano kit (Illumina, San Diego, CA), and amplicons were then sequenced by the Illumina MiSeq platform using the 2- by 300-bp paired-end V3 kit (Illumina).

**(ii) 16S sequencing data analysis.** Sequences were preprocessed, quality filtered, and analyzed using QIIME 2 (2019.10 release) (https://qiime2.org/) (49). QIIME 2 computes error-corrected amplicon sequence variants (ASV) for Illumina read sequences. We used QIIME 2 in combination with its Deblur plugin (50). Raw reads were imported into a QIIME 2 artifact before merging paired-end reads and quality filtering. Reads were then denoised using the “deblur denoise-16S” command, trimming reads at a length of 240 bases. Representative sequences and their abundances were extracted by feature-table (51). A naive Bayes classifier (52) was fitted with 16S rRNA gene sequences extracted from Greengenes version 13_8 (53). ASVs classified as from mitochondria or chloroplasts were excluded from further analysis. Compositions of microbiota communities were summarized by proportions at different taxonomy levels, including genus, family, order, class, and phylum ranks.

Beta diversity measures were calculated in QIIME 2, using UniFrac phylogenetic metrics (54). Principal-coordinate analyses were performed in QIIME 2 and visualized using ggplot2 (55) in R (56). Tests for categorical differences in beta diversity were performed using PERMANOVA (57) as implemented in R’s vegan package (58). Alpha diversity measures (observed ASV and Shannon diversity) were calculated from the ASV tables rarefied to 7,552 sequences per sample in QIIME 2. To identify the origin of the bacterial communities observed in samples collected on day 7 and day 14, we used SourceTracker (57). This probabilistic ASV-based algorithm employs an iterative Bayesian approach to predict which ASVs from pretreatment samples are likely to contribute to those in samples collected on day 7 and day 14.

### Statistical analysis.

Statistical analyses were performed with GraphPad prism software (version 8.0; GraphPad Software, San Diego, CA, USA). The results for each experimental group were evaluated by multivariate ANOVA, followed by a Bonferroni’s test to compare the groups two by two. A *P* value of <0.05 was considered to be statistically significant.

### Data availability.

The data set generated and analyzed during the current study is available in the NCBI repository under the primary accession number PRJNA674062.
